# Impact of cellular composition and T‐cell senescence of mononuclear cell concentrates on the manufacturing process of chimeric antigen receptor (CAR) T‐cells

**DOI:** 10.1111/trf.18354

**Published:** 2025-07-29

**Authors:** Vladan Vučinić, Theresa Tumewu, Mandy Brückner, Janine Kirchberg, Madlen Jentzsch, Raymund Buhmann, Yvonne Remane, Sandra Hoffmann, Florian Ramdohr, Maximilian Merz, Klaus H. Metzeler, Sebastian Schwind, Carmen Herling, Simon M. Krauß, Marco Herling, Georg‐Nikolaus Franke, Nora Grieb, Georg Stachel, Martin Janz, Olaf Penack, Lars Bullinger, Ulrich Keller, Michael Cross, Reinhard Henschler, Enrica Bach, Uwe Platzbecker

**Affiliations:** ^1^ Medical Clinic and Policlinic for Hematology, Cellular Therapy, Hemostaseology and Infectious Diseases Leipzig University Medical Center Leipzig Germany; ^2^ Comprehensive Cancer Center Central Germany Leipzig Germany; ^3^ Institute for Transfusion Medicine University Leipzig Medical Center Leipzig Germany; ^4^ Pharmacy University Leipzig Medical Center Leipzig Germany; ^5^ Innovation Center Computer Assisted Surgery (ICCAS) University Leipzig Leipzig Germany; ^6^ Medical Clinic and Policlinic for Cardiology Leipzig University Medical Center Leipzig Germany; ^7^ Campus Benjamin‐Franklin University Berlin Berlin Germany; ^8^ Campus Virchow‐Klinikum University Berlin Berlin Germany

**Keywords:** apheresis procedure, CAR T‐cells, out‐of‐specification events

## Abstract

**Background:**

Apheresis procedure of autologous lymphocytes competent for proliferation and expansion is a crucial step in the production of chimeric antigen receptor (CAR) T‐cells. Previous therapies or disease status prior to collection may negatively impact the collections.

**Study Design and Methods:**

We performed a retrospective analysis with the aim to determine cellular factors in association with the collection of autologous T‐cells and subsequent CAR T manufacturing toward tisagenlecleucel (tisa‐cel). Between February 2019 and February 2022, 63 collections of 54 patients were performed for subsequent therapy with tisa‐cel.

**Results:**

We observed no difference in median CD3+ cell yields according to the number of prior therapy lines (>3 vs. ≤3, *p* = .335), prior treatment with bendamustine (*p* = .954) or marrow infiltration (*p* = .634). Fifty‐six collections were sent for manufacturing, of which 22 (39%) resulted in manufacturing failures, namely terminations (*n* = 12) or out‐of‐specification events (*n* = 10).

Collections resulting in manufacturing failures yielded significantly lower CD3+ (*p* = .005), CD3+CD4+ (*p* = .044), and non‐senescent CD3+CD27+CD28+ (*p* = .003) counts. Multivariable analysis identified the absolute number of CD3+CD27+CD28+ cells as relevant, with a calculated cut‐off of ≥34.58 × 10^8^ CD3+CD27+CD28+ cells for 89.5% probability of successful CAR T‐cell production.

**Discussion:**

In summary, we report a positive influence of a higher number of non‐senescent Τ‐cells on successful manufacturing. Further analyses are required to determine measures for further optimization of collection outcomes.

**Abbreviations**
ALC: absolute lymphocyte count
ALL: acute lymphoblastic leukemia
CAR: chimeric antigen receptor
CR: complete remission
DLBCL: diffuse large B‐cell lymphoma
KLRG‐1: killer‐cell lectin‐like receptor subfamily G member 1
LAG‐3: lymphocyte activation gene‐3
OOS: out‐of specification
PD‐1: programmed cell death receptor 1
r/r: refractory or relapsed
RBC: red blood cells
TIM3: T‐cell immunoglobuline and mucin
tisa‐cel: tisagenlecleucel
WBC: white blood cells

## 1 INTRODUCTION

Chimeric antigen receptor (CAR) T‐lymphocytes have revolutionized the therapy of patients with B‐cell lymphoma, multiple myeloma, and B‐precursor acute lymphoblastic leukemia (ALL).^1^, ^2^, ^3^ Tisagenlecleucel (tisa‐cel, Novartis AG, Basel, Switzerland) is a CAR T product licensed for third‐line treatment of adult patients with follicular or aggressive B‐cell lymphoma as well as pediatric and young adolescent patients with refractory or relapsed (r/r) precursor B‐ALL.^4^, ^5^, ^6^, ^7^, ^8^

The complex manufacturing process of tisa‐cel starts with apheresis procedure of autologous T‐lymphocytes and cryoconservation at the apheresis site, followed by ex vivo viral transduction of the CAR T‐cell product and subsequent in vitro expansion at the manufacturing facility, taking altogether up to 4 weeks.^9^, ^10^, ^11^

According to the manufacturer's specifications for mononuclear cell concentrates, the requested target yield is ≥1.0 × 10^9^ CD3+ cells, with the values between 0.51 and 0.99 being rounded to 1.0 × 10^9^.^12^

Disease biology and prior treatment modalities can have influence on both the collection procedures as well as the outcome of collections of autologous lymphocytes resulting in insufficient CD3+ yield. Besides clinical complications which could postpone the timing of apheresis procedure and thus affect the timelines for production slots and manufacturing itself, a relevant number of patients have low white blood cell (WBC) counts, which may negatively influence the red blood cell (RBC)‐WBC interphase in the apheresis procedure device.^13^

Absolute lymphocyte count (ALC) and the number of circulating CD3+ cells in peripheral blood were identified as relevant factors influencing the collected yields of CD3+ cells^13^, ^14^ and can be negatively influenced by the nature and extent of prior anti‐lymphoma treatment.

Also, the intrinsic cellular properties of patients' CD3+ cells can be negatively affected, particularly states of exhaustion and senescence.^15^

Whereas the naïve and stem‐cell memory T‐cells show high expression of CD27+ and CD28+, the repetitive activation of T‐lymphocytes leads to a successive loss of costimulatory receptors CD27 and CD28^16^, ^17^ eventually resulting in non‐proliferating, senescent CD3+CD27−CD28− effector‐memory T‐cells.^18^ Besides the downregulation of CD27 and CD28, the phenotype of senescent T‐cells is characterized by high expression of CD57, killer‐cell lectin‐like receptor subfamily G member 1 (KLRG‐1), and CD45RA.^19^

We hypothesized that a predominant fraction of such non‐proliferating, senescent T‐cells in collections of autologous lymphocytes can have a negative impact on the manufacturing process of CAR T‐cells.

Therefore, we performed a retrospective analysis of patient‐associated factors influencing quantitative CD3+ yields of autologous mononuclear cell concentrates and successive manufacturing of tisa‐cel. Furthermore, we analyzed the cellular compositions of mononuclear cell concentrates with a particular focus on expression of CD27 and CD28 on CD3+ lymphocytes and their effect on the manufacturing process of CAR T‐cells. Moreover, we focused on the influence of the cellular composition of apheresis procedure collections on out‐of‐specification events (OOS) in a real‐world setting.

## 2 MATERIALS AND METHODS

### 2.1 Patients

Fifty‐four patients underwent collections of autologous lymphocytes for production of tisa‐cel between February 2019 and February 2022. The patients were a median 60.5 years old (range 17–80) and 41 (76%) were male.

To analyze the effect of age, patients were stratified into those <60 and ≥60 years. In total, 63 collections were performed after a median four treatment lines (range 1–7). All but one patient (diagnosed with ALL) had aggressive lymphomas. One patient underwent prophylactic apheresis procedure after the first treatment line. Forty‐eight patients underwent a single collection and one patient a 2‐day collection for the same production charge. Five patients had two collections and one patient three collections, but all were performed at different time points (no multi‐day collection).

The further demographic characteristics are presented in Table 1.

**TABLE 1:** Characteristics of patients undergoing lymphocyte apheresis procedure for production of tisagenlecleucel.

Patients	Total (*N* = 54)
Median age, years (range)	60.5 (17–80)
Age ≥60, *n* (%)	27/54 (50%)
Sex, male, *n* (%)	41/54 (76%)
Median body weight (kg), (range)	78 (42–113)
Lymphoma subtype, *n* (%)	
DLBCL	39 (72%)
t‐FL	12 (22%)
High‐grade	1 (2%)
B‐NHL NOS	1 (2%)
ALL	1 (2%)
Documented marrow infiltration, *n* (%)	12/52 (23%)
Number of previous treatment lines, *n* (%)	
1	1 (2%)
2	15 (28%)
3	12 (22%)
4	19 (35%)
5	6 (11%)
>5	1 (2%)
Prior treatment with bendamustine, *n* (%)	15/52 (30%)

Abbreviations: ALL, precursor acute lymphoblastic leukemia; B‐NHL NOS, B‐cell non‐Hodgkin lymphoma, not otherwise specified; DLBCL, diffuse large B‐cell lymphoma; t‐FL, transformed follicular lymphoma.

### 2.2 Assessment prior to apheresis procedure

Prior to apheresis procedure, all patients underwent an assessment of complete blood counts including WBC, ALC, and monocytes.

### 2.3 Apheresis procedure

All collections were performed with Spectra Optia device (Terumo BCT) using continuous MNC program, version 11 via peripheral venous lines. The apheresis procedure volume was set at three times the estimated blood volume, and collection time was limited to a maximum 5 h according to the national standards.^20^

Acid‐citrate‐dextrose was used as an anticoagulant at an inlet/outlet ratio of 12:1–15:1. Serum calcium levels were assessed routinely during apheresis procedure, and calcium was substituted intravenously as clinically indicated.

All collections were stored in cryopreservation media with 5% dimethyl sulfoxide according to previously published data^21^, ^22^ and preserved in the vapor phase of liquid nitrogen.

### 2.4 Evaluation of cell composition of collections

The determination of CD3+ cells in collections was performed on the day of apheresis procedure per flow cytometry as previously described.^23^ The target collection yield was arbitrarily defined as ≥0.55 × 10^9^ CD3+ cells.^12^ All collections underwent the additional determination of CD3+CD8+, CD3+CD4+ populations, as well as the CD4:CD8 ratio as previously described.^23^

Furthermore, all collections were assessed for CD27 and CD28 expression status on CD3+ cells.^24^

The expression of CD27 and CD28 in collections performed prior to February 1, 2020, was analyzed from previously cryoconserved aliquots of collections, while the analyses after February 1, 2020, were performed from patients' peripheral blood and fresh material on the day of collection.

### 2.5 Manufacturing

Fifty‐six (89%) cryoconserved mononuclear cell collections from 50 patients were shipped for CAR T manufacturing to Novartis facilities, resulting in successful manufacturing according to product specification (*n* = 34) or manufacturing failures (*n* = 22), of which 10 (45%) OOS and 12 (55%) terminations. All shipped mononuclear cell collections fulfilled the institutional specification requirements, the recommendations from Novartis, and were in line with national guidelines.^20^

### 2.6 Statistical analyses

The statistical analyses were performed by R software (version 3.6.2)^25^ and GraphPad Prism (version 10.3.1, GraphPad Software, Boston, Massachusetts USA). Data are presented as absolute numbers and percentages, medians, and ranges (defined as minimum–maximum). Continuous parameters and categorical variables were compared using the Kruskal–Wallis test and chi‐square test, respectively. Differences between groups were considered significant at *p*‐values <.05. The relation regarding cellular composition of peripheral blood and in collections was analyzed with logistic regression. We defined the receiver‐operating characteristics (ROC) curve for the CD3+CD27+CD28+ counts using the R proc. package.^26^ As a criterion for selecting the optimal cut‐off to discriminate between production success and failure, the Youden's index was calculated. In addition, we calculated the cut‐off for sensitivity >90% and specificity >90%. For each cut‐off, the positive predictive value (PPV) and negative predictive value (NPV) were calculated.

## 3 RESULTS

### 3.1 Parameters of peripheral blood prior to apheresis procedure

The median WBC prior to apheresis procedure was 5350/μL (range 1000–31,500; normal range: 3500–9800) and ALC 670/μL (range 140–5498; normal range: 1000–2900) with monocyte counts 875/μL (range 32–3780). The median absolute CD3+ count in peripheral blood was 697/μL (range 104–5220) with median CD3+CD27+CD28+ and CD3+CD27−CD28− counts of 237/μL (range 58–1369) and 224/μL (range 1–3088), respectively.

We noticed no differences in cell counts of peripheral blood according to patients' sex and age group (≤ vs. >60 years).

CD3+CD27+CD28+ counts in peripheral blood were 1.6‐fold lower in collections of patients having more than three treatment lines with 192/μL (range 75–547) versus 315/μL (range 58–1369; *p* = .008), 1.8‐fold lower in collections from patients with prior exposition to bendamustine with 198/μL (range 75–273) versus 352/μL (range 58–1369; *p* = .029) (Table 2).

**TABLE 2:** Clinical parameters and cellular composition of peripheral blood prior to apheresis procedure, *N* = 63.

Sex	Female	Male	*p*‐Value
WBC (/μL)	5700 (1200–22,500)	5300 (1000–31,500)	.694
ALC (/μL)	610 (320–1450)	730 (140–5498)	.476
Monocytes (/μL)	658 (98–1642)	966 (32–3780)	.070
CD3+ cells (/μL)	605 (228–906)	747 (104–5220)	.184
CD3+CD27+CD28+ cells (/μL)	207 (188–410)	288 (58–1369)	.529
CD3+CD27−CD28− cells (/μL)	53 (1–561)	279 (3–3088)	.294

Age	≤60 years	>60 years	*p*‐Value
WBC (/μL)	5800 (1900 – 31,500)	5300 (1000 – 14,000)	.279
ALC (/μL)	694 (140–5498)	670 (262–2500)	.552
Monocytes (/μL)	877 (156–3780)	875 (32–2507)	.736
CD3+ cells (/μL)	791 (104–5220)	686 (228–2031)	.502
CD3+CD27+CD28+ cells (/μL)	349 (58–1369)	210 (75–696)	.270
CD3+CD27−CD28− cells (/μL)	186 (5–3088)	225 (1–1706)	.565

Marrow infiltration	No	Yes	*p*‐Value
WBC (/μL)	5400 (1000–22,500)	5300 (2500 – 31,500)	.806
ALC (/μL)	630 (140–2500)	1125 (262–5498)	.031
Monocytes (/μL)	893 (99–3780)	630 (32–1325)	.107
CD3+ cells (/μL)	606 (104–2031)	929 (253–5220)	.043
CD3+CD27+CD28+ cells (/μL)	203 (58–696)	440 (190–1369)	.019
CD3+CD27−CD28− cells (/μL)	162 (1–1706)	288 (9–3088)	.247

Prior exposition to bendamustine	No	Yes	*p*‐Value
WBC (/μL)	5800 (1000–31,500)	4100 (1200–9800)	.103
ALC (/μL)	684 (140–5498)	560 (262–2500)	.016
Monocytes (/μL)	940 (32–3780)	702 (99–2336)	.131
CD3+ cells (/μL)	697 (104–5220)	688 (253–929)	.807
CD3+CD27+CD28+ cells (/μL)	352 (58–1369)	194 (75–257)	.029
CD3+CD27−CD28− cells (/μL)	77 (1–3088)	281 (9–654)	.310

Previous treatment lines	≤3	>3	*p*‐Value
WBC (/μL)	5800 (1000–31,500)	5300 (1900–14,000)	.446
ALC (/μL)	969 (237–5498)	587 (140–2500)	.092
Monocytes (/μL)	858 (32–1984)	963 (156–3780)	.354
CD3+ cells (/μL)	854 (104–5220)	593 (272–2031)	.335
CD3+CD27+CD28+ cells (/μL)	353 (58–1369)	192 (75–547)	.008
CD3+CD27−CD28− cells (/μL)	238 (1–3088)	208 (3–1706)	.999

Abbreviations: ALC, absolute lymphocyte count; WBC, white blood cells.

### 3.2 Collections

The median yield of CD3+ cells was 48.6 (range 0.4–320) × 10^8^, with yields for CD3+CD4+ and CD3+CD8+ of 20.1 (range 2.4–102.7) × 10^8^ and 27.4 (range 3.8–266.8) × 10^8^, respectively, resulting in a median CD4:CD8 ratio of 0.7 (range 0.1–5.6).

**FIGURE 1:**
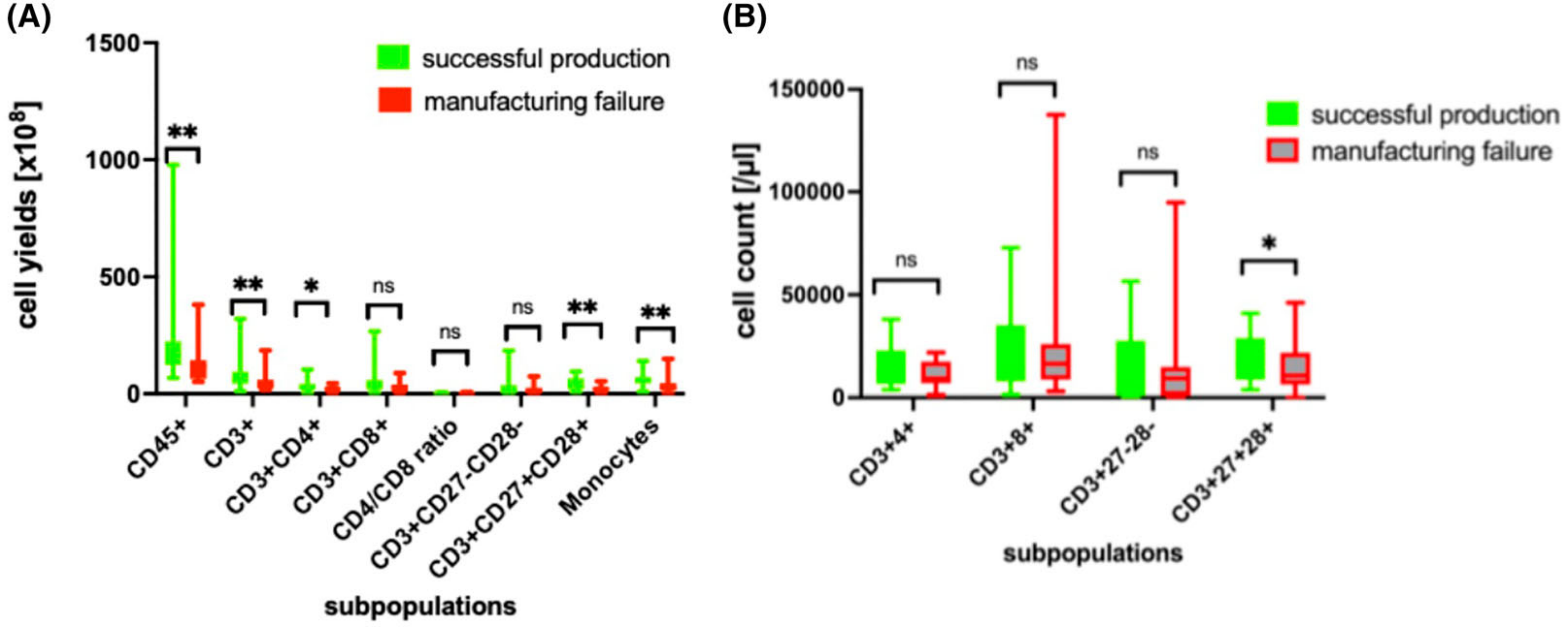
Cellular composition of graft and manufacturing of chimeric antigen receptor T‐cells. (A) Collected yields (absolute numbers), (B) concentrations of cells (cells/μL) in collections. *p < .05 and **p < .01. [Color figure can be viewed at wileyonlinelibrary.com]

Furthermore, median yields for CD3+CD27+CD28+ and CD3+CD27−CD28− cells were 25.9 (range 1.1–145.3) × 10^8^ and 10.3 (range 0.1–183.8) × 10^8^, respectively. The cellular composition of peripheral blood and mononuclear cell concentrates is presented in Figure 1.

We found a correlation between the CD3+ cells in peripheral blood and collected CD3+ yields (*r* = 0.87, *p* < .01).

### 3.3 Impact of patient‐related factors on cell composition

We observed no difference in CD3+ yields according to sex (*p* = .583), age group (*p* = .622), number of prior treatments (*p* = .335), prior treatment with bendamustine (*p* = .954) or marrow infiltration (*p* = .634), Table 3.

**TABLE 3:** Cellular composition of lymphocyte concentrates, *N* = 63.

Sex	Female	Male	*p*‐Value
CD45+ (×10^8^)	122.4 (52.1–334.30)	141.9 (4.7–977.9)	.230
Monocytes (×10^8^)	43.1 (9.9–118.8)	46.9 (0.1–148.6)	.424
CD3+ (×10^8^)	42.1 (15.8–162.6)	51.3 (4.1–319.8)	.583
CD3+CD4+ (×10^8^)	19.4 (3.1–102.7)	20.3 (2.4–80.3)	.891
CD3+CD4+ (/μL)	8750 (2048–38,054)	9986 (1037–36,661)	.504
CD3+CD8+ (×10^8^)	35.6 (4.2–118.5)	26.6 (2.4–266.8)	.571
CD3+CD8+ (/μL)	15,295 (2263–43,100)	13,293 (1395–137,517)	.959
CD4:CD8 ratio (1:1)	0.72 (0.2–4.0)	0.62 (0.1–5.6)	.771
CD3+CD27+CD28+ (×10^8^)	25.3 (1.1–79.7)	25.9 (3.3–145.3)	.864
CD3+CD27+CD28+ (/μL)	11,723 (763–28,967)	11,446 (14–78,536)	.918
CD3+CD27−CD28− (×10^8^)	13.9 (0.1–91.1)	10.2 (0.2–183.8)	.904
CD3+CD27−CD28− (/μL)	5643 (25–34,403)	4259 (78–94,743)	.864

Age	≤60	>60	*p*‐Value
CD45+ (×10^8^)	141.3 (4.7–977.9)	135.7 (50.6–313.5)	.948
Monocytes (×10^8^)	48.3 (0.1–148.5)	44.4 (4.1–137.4)	.994
CD3+ (×10^8^)	46.6 (4.1–319.8)	48.6 (19.3–192.3)	.622
CD3+CD4+ (×10^8^)	20.1 (2.4–63.5)	20.1 (5.7–102.7)	.908
CD3+CD4+ (/μL)	9599 (1037–25,386)	9867 (2440–38,054)	.684
CD3+CD8+ (×10^8^)	36.4 (3.8–266.8)	26.1 (3.8–164.1)	.186
CD3+CD8+ (/μL)	21,289 (1395–137,517)	13,282 (1425–72,946)	.212
CD4:CD8 ratio (1:1)	0.54 (0.2–2.6)	1 (0.1–5.6)	.565
CD3+CD27+CD28+ (×10^8^)	26.4 (1.1–145.3)	24.3 (7.8–89.7)	.710
CD3+CD27+CD28+ (/μL)	12,802 (14–78,536)	11,210 (3724–40,967)	.954
CD3+CD27−CD28− (×10^8^)	7.7 (0.2–183.8)	11.9 (0.1–110.9)	.994
CD3+CD27−CD28− (/μL)	4097 (78–94,743)	5132 (25–56,602)	.999

Marrow infiltration	No	Yes	*p*‐Value
CD45+ (×10^8^)	141.3 (4.7–380.4)	120.8 (50.5–977.9)	.888
Monocytes (×10^8^)	52.7 (0.1–148.5)	29.6 (4.1–69.1)	.**038**
CD3+ (×10^8^)	48.6 (4.1–192.3)	46.6 (15.8–319.8)	.634
CD3+CD4+ (×10^8^)	19.8 (2.4–102.7)	25.9 (3.1–60.1)	.386
CD3+CD4+ (/μL)	9097 (1037–38,054)	14,946 (2048–22,950)	.069
CD3+CD8+ (×10^8^)	25.9 (3.8–164.1)	36.4 (4.2–266.8)	.292
CD3+CD8+ (/μL)	13,272 (1394–72,946)	21,902 (2854–137,517)	.092
CD4:CD8 ratio (1:1)	0.7 (0.1–5.3)	0.7 (0.2–5.6)	.551
CD3+CD27+CD28+ (×10^8^)	22.3 (3.3–93.5)	29.0 (1.1–145.3)	.165
CD3+CD27+CD28+ (/μL)	9737 (3047–40,967)	16,804 (14–78,536)	.075
CD3+CD27−CD28− (×10^8^)	9.4 (0.1–110.9)	10.8 (0.7–183.8)	.654
CD3+CD27−CD28− (/μL)	4097 (78–94,743)	6698 (326–94,743)	.310

Prior exposition to bendamustine	No	Yes	*p*‐Value
CD45+ (×10^8^)	141.1 (4.7–977.9)	135.4 (51.9–380.4)	.653
Monocytes (×10^8^)	48.3 (0.1–139.4)	41.9 (7.8–148.5)	.829
CD3+ (×10^8^)	49.9 (4.1–319.8)	46.4 (15.8–319.2)	.954
CD3+CD4+ (×10^8^)	21.8 (2.4–102.7)	17.0 (3.1–62.9)	.254
CD3+CD4+ (/μL)	10,450 (1037–38,054)	9483 (2048–23,728)	.288
CD3+CD8+ (×10^8^)	25.9 (3.8–164)	36.4 (2.4–266.8)	.300
CD3+CD8+ (/μL)	13,283 (1426–137,517)	15,631 (1395–63,317)	.251
CD4:CD8 ratio (1:1)	0.7 (0.1–5.3)	0.6 (0.2–5.6)	.364
CD3+CD27+CD28+ (×10^8^)	27.1 (3.3–145.3)	18.9 (1.1–76.1)	.160
CD3+CD27+CD28+ (/μL)	14,095 (3047–78,536)	9637 (14–28,700)	.070
CD3+CD27−CD28− (×10^8^)	6.8 (0.1–183.8)	17.3 (0.6–120.0)	.383
CD3 + CD27−CD28− (/μL)	3450 (25–94,743)	7908 (218–49,376)	.472

Prior treatment lines	≤3	>3	*p*‐Value
CD45+ (×10^8^)	135.3 (50.5–977.9)	141.1 (4.7–380.4)	.977
Monocytes (×10^8^)	37.7 (4.1–139.4)	57.1 (0.1–148.5)	.065
CD3+ (×10^8^)	56.6 (9.7–319.8)	43.6 (4.1–185.1)	.335
CD3+CD4+ (×10^8^)	25.1 (2.6–102.7)	17.8 (2.4–63.5)	.065
CD3+CD4+ (/μL)	12,205 (1221–38,054)	7673 (1307–25,386)	.011
CD3+CD8+ (×10^8^)	27.4 (4.7–266.8)	27.1 (3.8–153.9)	.818
CD3+CD8+ (/μL)	13,744 (2263–137,517)	13,900 (1395–63,317)	.614
CD4:CD8 ratio (1:1)	0.7 (0.1–5.6)	0.5 (0.2–5.3)	.379
CD3+CD27+CD28+ (×10^8^)	32.4 (6.6–145.3)	18.2 (1.1–93.5)	.**003**
CD3+CD27+CD28+ (/μL)	16,362 (3047–78,536)	8099 (14–37,406)	**<.001**
CD3+CD27−CD28− (×10^8^)	10.3 (0.1–183.8)	11.1 (0.3–120)	.639
CD3+CD27−CD28− (/μL)	5053 (25–94,743)	4547 (87–56,602)	.795

Patients with more than three prior treatment lines had 1.78‐fold lower CD3+CD27+CD28+ yields with 18.2 (range 1.1–93.5) × 10^8^ comparing to 32.4 (range 6.6–145.3) × 10^8^ (*p* < .01).

The influence of clinical factors on cell compositions of mononuclear cell concentrates is presented in Table 3.

### 3.4 Influence of clinical factors and cell composition of peripheral blood on manufacturing success

Thirty‐four of 56 (61%) collections resulted in successful manufacturing according to the product specification, whereas the remaining 22 collections (39%) from 17 patients resulted in manufacturing failure, with 10/22 OOS and 12/22 terminations.

Reasons for manufacturing failures were insufficient proliferation or expansion of CAR T‐cells (*N* = 16), microbiological contamination (*N* = 1) or undeterminable testing of mycoplasma contamination after viral transduction (*N* = 5), respectively.

We did not notice differences in the occurrence of manufacturing failures according to assessed clinical factors (Table 4).

**TABLE 4:** Clinical factors and production success.

Collections	Succesful manufacturing	Manufacturing failure	Total	*p*‐Value
Male sex	27/34	16/22	43	.563
Age ≥ 60	17/34	15/22	32	.179
>3 prior treatment lines	18/34	10/22	28	.584
Bendamustine^a^	8/32	10/22	18	.117
Marrow infiltration^b^	6/33	5/20	11	.553

^a^ Prior exposition to bendamustine not known for two collections.

^b^ Marrow infiltration for three collections not known.

ALC and CD3+ concentration in peripheral blood determined prior to apheresis procedure did not differ between collections resulting in successful productions or manufacturing failures (see Table S1).

### 3.5 The influence of cell composition in collections on the manufacturing process

Total collected cell count in successful productions was on average 1.49‐fold higher than in manufacturing failures with median 173.10 (range 66.27–977.9) × 10^8^ and 116.0 (range 50.5–380.4) × 10^8^ respectively (*p* < .001). The median CD3+ cell yield in successful productions was 1.62‐fold higher with 57.9 (range 9.7–320) × 10^8^ compared to 35.8 (range 15.8–185) × 10^8^ in manufacturing failures (*p* = .005). The median CD3+CD4+ yield in successful productions was 1.31‐fold higher than in manufacturing failures, with 25.4 (range 2.6–102.7) × 10^8^ versus 19.4 (range 3.1–43.4) × 10^8^, respectively (*p* = .044), whereas the yields for CD3+CD8+ cells showed trend for higher values in successful productions (*p* = .085).

**FIGURE 2:**
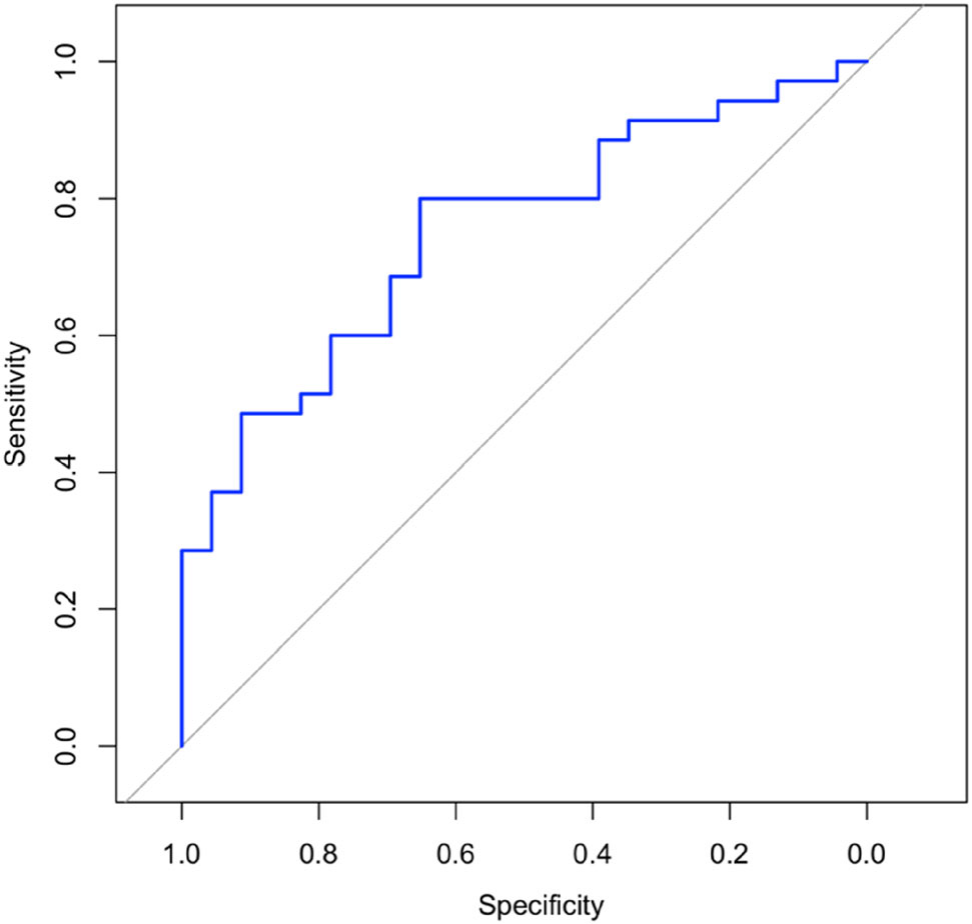
Receiver‐operating characteristic curve for the prediction of manufacturing success based on CD3+CD27+CD28+ collection yields. The area under the curve is 0.7338 (95% confidence interval: 0.6041–0.8635). [Color figure can be viewed at wileyonlinelibrary.com]

While the CD27−CD28− yields showed no difference (*p* = .700), the median CD27+CD28+ yield was 1.81‐fold higher in successful productions with 31.41 (range 6.55–93.52) × 10^8^ compared to 17.34 (range 1.13–52.29) × 10^8^ (*p* = .003) in manufacturing failures (Figure 2).

The univariate analysis showed the influence of higher yields of CD45+ cells (*p* = .023), monocyte yields (*p* = .011), yields of CD3+ cells (*p* = .046), CD3+CD27+CD28+ yields (*p* = .009), CD3+CD4+ cells (*p* = .031) as well as concentrations of CD3+CD8+ cells (*p* = .031) and concentrations of CD3+CD27+CD28+ cells in the collections (*p* = .027) resulting in successful manufacturing (see Table S2).

However, in multivariable analysis only the yields of CD3+CD27+CD28+ were defined as independent predictors of successful manufacturing (OR 0.823, 95% confidence interval 0.698–0.971, *p* = .021) (see Table S3).

### 3.6 The relationship between CD3+CD27+CD28+ values and manufacturing success

The ROC curve illustrating the relationship between CD3+CD27+28+ values and manufacturing success is presented in Figure 2, with corresponding coordinates documented in Table S4. The optimal cut‐off point, based on Youden's index, was determined to be 34.58 × 10^8^. This resulted in a PPV of 0.895 and a NPV of 0.526. Consequently, patients with CD27+CD28+ yields >34.58 × 10^8^ had successful production in 89.5% of cases, while patients with CD27+CD28+ values ≤34.58 × 10^8^ encountered manufacturing failure in 52.6% of cases.

The cellular composition of successful productions and manufacturing failures is presented in Table 5. The composition of insufficient cell proliferation and other reasons is presented in Table S5.

**TABLE 5:** Cellular composition of successful productions and manufacturing failures.

Cellular population	Successful manufacturing	Manufacturing failure	*p*‐Value
Peripheral blood			
Monocytes (/μL)	963 (99–2507)	496 (32–2336)	.**016**
WBC (/μL)	6000 (1200–31,500)	4800 (1000–13,700	.134
ALC (/μL)	730 (140–5498)	622.5 (252–2514)	.115
CD3 (/μL)	822.6 (104–5220)	640 (228–2426)	.312
Collections			
CD45+ (×10^8^)	173.1 (66.3–977.9)	116 (50.5–380.4)	.**002**
Monocytes (×10^8^)	60.3 (7.8–139.4)	24.8 (4.1–148.5)	**<.001**
CD3+ (×10^8^)	58 (10–320)	36 (16–185)	.**005**
CD3+CD4+ (×10^8^)	25.4 (2.6–102.7)	19.4 (3.1–43.4)	.**044**
CD3+CD8+ (×10^8^)	34.9 (3.9–266.8)	25.7 (3.8–87.3)	.085
CD3+CD27+CD28+ (×10^8^)	31.4 (6.6–93.5)	17.3 (1.1–52.3)	.**003**
CD3+CD27−CD28− (×10^8^)	10.9 (0.2–183.8)	11.9 (0.1–73.3)	.700
CD4:CD8 ratio	0.6 (0.1–5.3)	0.7 (0.2–5.6)	.566
CD3+CD4+ (/μL)	11,230 (1221–38,050)	9348 (2048–20,750)	.154
CD3+CD8+ (/μL)	15,630 (1426–137,500)	12,440 (1395–40,980)	.195
CD3+CD27+CD28+ (/μL)	14,710 (14–46,080)	9761 (763–26,230)	.**042**
CD3+CD27−CD28− (/μL)	6076 (78–94,740)	5132 (25–34,400)	.909

Abbreviations: ALC, absolute lymphocyte count; WBC, white blood cell.

Nine OOS products (six with indeterminable mycoplasma test and three with insufficient cell counts) were returned. No detection of mycoplasma infection in further follow‐up was recorded.

## 4 DISCUSSION

We performed a retrospective analysis of 63 collections of autologous lymphocytes intended for the manufacturing of tisa‐cel.

Our collections yielded a median of 46.7 × 10^8^ CD3+ cells with a single collection in all but one patient.

Manufacturing issues were one of the main reasons for patients' exclusion from clinical trials with CAR T‐cells. In a Phase II global trial of tisa‐cel treatment of adult patients with aggressive lymphomas (JULIET), which included diffuse large B‐cell lymphoma (DLBCL) patients after more than two previous treatment lines, 12 of 165 enrolled patients could not have CAR T‐cells manufactured and were excluded.^4^

Also, in patients with aggressive B‐cell lymphoma treated in earlier lines, for example, in a “Phase 3 trial of second‐Line tisagenlecleucel or standard care in aggressive B‐cell lymphoma” (BELINDA), manufacturing issues were reported in one of 135 patients.^27^ In contrast to clinical trials, the real‐world settings allow for multiple aphereses procedures and manufacturing attempts of the same patient, thus resulting in higher percentages of OOS.

Reports on mononuclear cell concentrates for CAR T manufacturing are predominantly limited to the pediatric population, mostly patients with ALL or neuroblastoma, and are focused on reaching the target count of CD3+ yields. Allen et al. reported that 55 of 71 pediatric collections of patients with ALL, neuroblastoma, and lymphoma reached the arbitrarily defined target of 2 × 10^9^ CD3+ cells.^13^

Recently, Even‐Or et al. reported on 26 collections of autologous lymphocytes of 20 pediatric ALL patients. In all but one collection, the target yield of 0.9 × 10^9^ CD3+ cells could be reached. Interestingly, this group compared the collection efficiency between Cobe Spectra and Spectra Optia devices and confirmed the comparability of both approaches.^28^

Available reports about collections from adult patients are in line with pediatric data. Harrer et al. reported about 24 collections of 23 adult patients with B‐cell lymphoma with the target criterion of 1 × 10^9^ CD3+ cells met in 21 patients.^14^ In a Japanese national trial including 408 patients, Yo et al. reported manufacturing failures in 7.4% of cases.^29^ Furthermore, they identified a significantly higher proportion of patients with prior bendamustine treatment as well as a significantly lower CD4:CD8 ratio in peripheral blood prior to apheresis procedure among patients with manufacturing failure.

Sufficient ALC in peripheral blood is one of the most relevant factors for reaching the appropriate CD3+ yield and planning of collections.^30^, ^31^ Also, in our cohort, a correlation of CD3+ cells in peripheral blood and collected CD3+ cells was demonstrated.

Another relevant issue for the manufacturing of CAR T‐cells is the percentage of monocytes in leukapheresis material. Monocytes and macrophages in the collections are considered contaminants because of their potential to digest the antibody‐conjugated beads, which are used for CAR T manufacturing.^31^, ^32^ The results in our cohort were not in line with this hypothesis, as both the absolute monocyte count in the peripheral blood of patients with successful productions and the monocyte yields were significantly higher compared to manufacturing failures. The role of monocytes in apheresis procedure material intended for the manufacturing process of CAR T‐cells should further be explored.

In our cohort of 22 manufacturing failures, six showed an indeterminable mycoplasma test during the manufacturing of CAR T‐cells, as required per specification.^33^ As no deviations in processes during the apheresis procedure or cryoconservation of those products were detected, we cannot exclude that the preanalytic tests in manufacturing facilities influenced this issue. All those products were returned without problems. After excluding the OOS issues due to indeterminate mycoplasma test, OOS incidence in our dataset would be in line with recently published data of Worel et al. with 5.9% cumulative incidence for tisa‐cel.^34^

Iacobonni et al. addressed the frequently discussed question of bendamustine application prior to apheresis procedure. Four‐hundred and thirty‐nine patients with large B‐cell lymphoma treated with CAR T‐cells in the commercial setting were included, 80 of whom had prior exposition to bendamustine. The CD3+ count prior to apheresis procedure in this group was significantly lower. Interestingly, bendamustine exposed patients had shorter progression‐free survival and shorter overall survival.^35^ The effects of bendamustine on lymphocytes are lasting leukopenias and low CD4+ counts for 7–9 months as well as negative effects on proliferation of CD3+ cells.^36^ This aspect focuses on the issue of bridging therapies prior to CAR T therapy. Currently, one of the most frequently used treatments for bridging prior to CAR T therapy is the combination of polatuzumab‐vedotin, bendamustine, and rituximab.^37^

Our data, albeit limited by the small number of patients, could not demonstrate the influence of prior treatment with bendamustine on the manufacturing process. Further analyses in larger patient cohorts are necessary to address this issue. However, in concordance with published findings, in patients exposed to bendamustine, we detected lower counts of non‐senescent CD3+CD27+CD28+ cells in peripheral blood.

We also addressed the functional characteristics of collected CD3 cells by determining senescence parameters CD27 and CD28 and their effects on CAR T manufacturing. Our findings of lower counts of CD3+CD27+CD28+ cells in peripheral blood and in collections of patients with more than three treatment lines are in line with these observations.

Noteworthy, compared to manufacturing failures, successful productions in our cohort showed significantly higher yields of all cell‐subpopulations examined except CD3+CD27−CD28−.

The influence of the expression of CD27 and CD28 in autologous collections of CD3+ lymphocytes on the outcome of patients successively treated with CAR T‐cells was recently published. Worel et al. identified the low frequency of CD3+CD27−CD28− cells in peripheral blood at apheresis procedure as a favorable factor for overall survival and likelihood for complete remission (CR). Furthermore, the proliferation of CAR T‐cells in patients and their cytotoxic cytokine production was higher in the collections with CD3+CD8+CD27+CD28+ phenotype compared to CD3+CD8+CD27−CD28− cells.^24^ Similarly, Cuffel et al. reported in a retrospective analysis of 30 patients with aggressive B‐cell lymphoma that the predominantly naïve and stem‐cell memory phenotype of T‐cells in mononuclear cell collections shows a strong impact on both the early and persisting response after CAR T treatment with tisa‐cel for aggressive B‐cell lymphoma.^38^

This issue is also relevant in the context of future treatment developments. In the light of the emerging use of CD3+ cells activating bispecific antibodies^39^, ^40^ and their potential application in treatment lines prior to CAR T‐cells, the markers of T‐cell exhaustion and senescence will gain clinical relevance. Bispecific antibodies could functionally affect the T‐lymphocytes of the patients, causing senescence and exhaustion^15^ and thus have a negative impact on the manufacturing process of CAR T‐cells.

Exhausted T‐lymphocytes show impaired cytotoxicity, expression of inhibitory receptors such as programmed cell death receptor 1 (PD‐1), T‐cell immunoglobulin and mucin domain containing‐3 (Tim‐3), and lymphocyte activation gene‐3 (LAG‐3). In contrast to senescence being an irreversible process, the exhaustion of T‐lymphocytes can be reversed with check‐point blockade.^15^

Collections of our patients resulting in manufacturing failures had significantly lower total CD3+ counts, as well as lower CD3+CD4+ and CD3+CD27+CD28+ counts. Especially, the counts of CD3+27+28+ cells confirm the negative influence of T‐cell senescence on the manufacturing process. Unfortunately, the analyses of cellular composition were restricted to CD27 and CD28 without including further senescence markers, like CD57, KLRG‐1, and CD45RA.^19^ We recommend the analyses of further senescence and exhaustion markers of lymphocyte concentrates, but also a comparison of the cellular composition of mononuclear cell concentrates and manufactured CAR T products.

Our ROC curve analysis demonstrated the cut‐off of 34.58 × 10^8^ CD3+CD27+CD28+ cells in collections for 89.5% probability of successful production rate of CAR T‐cells. These data implicate that the mononuclear cell concentrates for production of CAR T‐cells should be performed at earlier stages of treatment.

Our analysis has several limitations. First, it has a retrospective character and is restricted to mononuclear cell collections for tisa‐cel. Furthermore, the CAR T‐cell manufacturing process can be influenced by further relevant factors like culture media, cytokines, and culture period. However, this analysis emphasizes the relevance of the cellular composition of starting material regarding manufacturing issues, and especially OOS events as a relevant challenge in the treatment with CAR T‐cells. Further prospective trials, especially trials with a focus on the functional fitness of T‐cells, are necessary to further address this relevant issue.

## AUTHOR CONTRIBUTIONS

Conception and design: **Vladan Vučinić**, **Uwe Platzbecker**. Acquisition of data: **Vladan Vučinić**, **Theresa Tumewu**, **Mandy Brückner**, **Florian Ramdohr**, **Janine Kirchberg**, **Sandra Hoffmann**, **Martin Janz**, **Olaf Penack**, **Michael Cross**, **Enrica Bach**. Analysis and interpretation of data: **Vladan Vučinić**, **Theresa Tumewu**, **Georg Stachel**, **Nora Grieb**, **Simon M. Krau**ß. Administrative support: **Madlen Jentzsch**, **Raymund Buhmann**, **Yvonne Remane**, **Maximilian Merz**, **Klaus H. Metzeler**, **Sebastian Schwind**, **Carmen Herling**, **Marco Herling**, **Georg‐Nikolaus Franke**, **Martin Janz**, **Olaf Penack**, **Lars Bullinger**, **Ulrich Keller**, **Reinhard Henschler**. Writing, review, and revision of the manuscript: all authors.

## CONFLICT OF INTEREST STATEMENT

VV receives honoraria from Novartis, Gilead/Kite, J&J, BMS/Celgene and travel grants from Gilead/Kite and J&J. MJ receives honoraria from Novartis, BMS/Celgene, J&J and travel grants from J&J. G‐NF receives honoraria from Novartis, Gilead/Kite, J&J, BMS Celgene and travel grants from Gilead/Kite and J&J. MM receives honoraria from Gilead/Kite, J&J, BMS/Celgene, travel grants and research support from J&J. All other authors have nothing to disclose.

## ETHICS STATEMENT

This analysis was approved by the ethics committee of the University of Leipzig (approval number: 289/23‐ek) and all participants gave written informed consent.

## Supporting information


**Data S1.** Supporting Information.

## Data Availability

The datasets generated during and/or analyzed during the current study are available from the corresponding author on reasonable request.
