# Supplementary material for: Impact of cellular composition and T‐cell senescence of mononuclear cell concentrates on the manufacturing process of chimeric antigen receptor (CAR) T‐cells
Source: Transfusion. 2025 Jul 29;65(9):1650–61. doi: 10.1111/trf.18354 (PMC12432817; doi:10.1111/trf.18354)
Supplement: Supplementary file 1 — Data S1. Supporting Information. [file TRF-65-1650-s001.docx]

Impact of Cellular Composition and T-Cell Senescence of Lymphocyte Collections to the Manufacturing Process of Chimeric Antigen Receptor (CAR) T-Cells

Vučinić et al.

Supplemental Table 1. Cellular composition of peripheral blood and yields resulting in successful productions and manufacturing failures

| **Cellular population** | **successful manufacturing** | **manufacturing failure** | **p-value** |
| --- | --- | --- | --- |
|  |  |  |  |
| **Peripheral blood** |  |  |  |
| Monocytes [/µl] | 963 (99 – 2,507) | 496 (32 – 2,336) | **0.016** |
| WBC [/µl] | 6,000 (1,200 – 31,500) | 4,800 (1,000 – 13,700 | 0.134 |
| ALC [/µl] | 730 (140 – 5,498) | 622.5 (252 – 2,514) | 0.115 |
| CD3 [/µl] | 822.6 (104 – 5,220) | 640 (228 – 2,426) | 0.312 |
|  |  |  |  |
| **Collections** |  |  |  |
| CD45+ cells [x10^8^] | 173.1 (66.3 – 977.9) | 116 (50.5 – 380.4) | **0.002** |
| Monocytes [x10^8^] | 60.3 (7.8 – 139.4) | 24.8 (4.1 – 148.5) | **<0.001** |
|  |  |  |  |
| CD3 yield [x10^8^] | 58 (10 – 320) | 36 (16 – 185) | **0.005** |
| CD3+4+ [x10^8^] | 25.4 (2.6 – 102.7) | 19.4 (3.1 – 43.4) | **0.044** |
| CD3+CD8+ [x10^8^] | 34.9 (3.9 – 266.8) | 25.7 (3.8 – 87.3) | 0.085 |
| CD3+27+28+ [x10^8^] | 31.4 (6.6 – 93.5) | 17.3 (1.1 – 52.3) | **0.003** |
| CD3+27-28- [x10^8^] | 10.9 (0.2 – 183.8) | 11.9 (0.1 – 73.3) | 0.700 |
|  |  |  |  |
| CD4:CD8 ratio | 0.6 (0.1 – 5.3) | 0.7 (0.2 – 5.6) | 0.566 |
|  |  |  |  |
| CD3+4+ [/µl] | 11230 (1221 – 38,050) | 9,348 (2048 – 20750) | 0.154 |
| CD3+8+ [/µl] | 15,630 (1,426 – 137,500) | 12,440 (1,395 – 40,980) | 0.195 |
| CD3+27+28+ [/µl] | 14,710 (14 – 46,080) | 9,761 (763 – 26,230) | 0.042 |
| CD3+27-28- [/µl] | 6,076 (78 – 94,740) | 5,132 (25 – 34,400) | 0.909 |

Supplemental Table 2. Univariable analysis demonstrating the influence of cell composition and clinical factors to successful manufacturing

| category | OR (95% CI) | p-value |
| --- | --- | --- |
| ALC prior to Apheresis | 1.000 (1.000 - 1.001) | 0.355 |
| CD3+ pB | 1.000 (0.999 - 1.002) | 0.472 |
| CD3+27-CD28- PB[/µl] | 1.001 (0.999 - 1.002) | 0.414 |
| CD3+CD27+28+ PB[/µl] | 1.001 (0.998 - 1.005) | 0.491 |
| CD3+ yield [x10^8^] | 1.018 (1.000 - 1.037) | **0.046** |
| CD45 [x10^8^] | 1.010 (1.001 - 1.019) | **0.023** |
| CD3+27-28- collections [/µl] | 1.000 (1.000 – 1.000) | 0.357 |
| CD3+27+28+ collections [/µl] | 1.000 (1.000 - 1.000) | **0.027** |
| CD4:CD8 ratio | 0.913 (0.627 - 1.329) | 0.632 |
| CD3+4+ collections [/µl] | 1.000 (1.000 - 1.000) | 0.076 |
| CD3+4+ yield [x10^8^] | 1.049 (1.004 - 1.096) | **0.031** |
| CD3+CD8+ collections[/µl] | 1.049 (1.004 - 1.096) | **0.031** |
| CD3+8+ yield [x10^8^] | 1.018 (0.998 - 1.038) | 0.077 |
| CD3+27-28- yield [x10^8^] | 1.011 (0.993 - 1.029) | 0.248 |
| CD3+27+28+ yield [x10^8^] | 1.055 (1.014 - 1.098) | **0.008** |
| Monocytes yield [x10^8^] | 1.028 (1.006 - 1.050) | **0.011** |
| Age >60 years | 0.971 (0.927 - 1.017) | 0.219 |
| Sex | 1.266 (0.372 - 4.310) | 0.706 |
| >3 treatment lines | 1.242 (0.771 - 2.000) | 0.374 |
| Prior bendamustine | 0.450 (0.144 - 1.403) | 0.168 |
| Prior bone-marrow infiltration | 0.643 (0.168 - 2.463) | 0.519 |

Supplemental Table 3. Multivariable analysis demonstrating the influence of cell composition to successful manufacturing.

|  | OR (95% CI) | p-value |
| --- | --- | --- |
| CD3+ yield [x10^8^] | 0.988 (0.956 – 1.021) | 0.485 |
| CD45 yield [x10^8^] | 1.003 (0.990 – 1.017) | 0.621 |
| CD3+27+28+ collections [/µl] | 1.000 (0.999 – 1.000) | 0.083 |
| CD3+4+ yield [x10^8^] | 0.976 (0.925 – 1.028) | 0.351 |
| CD3+27+28+ yield [x10^8^] | 1.216 (1.030 – 1.432) | **0.021** |
| Monocytes yield [x10^8^] | 1.014 (0.990 – 1.040) | 0.259 |

Supplemental Table 4. Receiver operating curve – table with calculated cut-off for specificity >90% and sensitivity >90%

| **Threshold CD3+27+28+[10^8^]** | **specificity** | **sensitivity** |
| --- | --- | --- |
| 3.84380005 | 0.045454545 | 1 |
| 7.16 | 0.045454545 | 0.971428571 |
| 8.8382259 | 0.090909091 | 0.971428571 |
| 9.9182259 | 0.090909091 | 0.942857143 |
| 10.078773 | 0.136363636 | 0.942857143 |
| 10.4194475 | 0.181818182 | 0.942857143 |
| 11.0056745 | 0.181818182 | 0.914285714 |
| 11.6893837 | 0.227272727 | 0.914285714 |
| 12.2393837 | 0.272727273 | 0.914285714 |
| 13.05 | 0.318181818 | 0.914285714 |
| 13.78 | 0.318181818 | 0.885714286 |
| 14.405 | 0.363636364 | 0.885714286 |
| 15.03 | 0.363636364 | 0.857142857 |
| 15.5115128 | 0.363636364 | 0.828571429 |
| 15.8746856 | 0.363636364 | 0.8 |
| 16.0424071 | 0.409090909 | 0.8 |
| 16.7292343 | 0.454545455 | 0.8 |
| 17.34 | 0.5 | 0.8 |
| 17.5116527 | 0.545454545 | 0.8 |
| 18.2229812 | 0.590909091 | 0.8 |
| 18.8413285 | 0.590909091 | 0.771428571 |
| 19.285 | 0.590909091 | 0.742857143 |
| 20.2928112 | 0.590909091 | 0.714285714 |
| 21.6278112 | 0.590909091 | 0.685714286 |
| 22.635879 | 0.636363636 | 0.685714286 |
| 23.5404329 | 0.636363636 | 0.657142857 |
| 24.8745539 | 0.636363636 | 0.628571429 |
| 25.725 | 0.681818182 | 0.628571429 |
| 26.115 | 0.681818182 | 0.6 |
| 26.5473282 | 0.727272727 | 0.6 |
| 26.9523282 | 0.772727273 | 0.6 |
| 28.105 | 0.772727273 | 0.571428571 |
| 29.9412834 | 0.772727273 | 0.542857143 |
| 31.0299277 | 0.772727273 | 0.514285714 |
| 31.3086443 | 0.818181818 | 0.514285714 |
| 31.9390613 | 0.818181818 | 0.485714286 |
| 33.3090613 | 0.863636364 | 0.485714286 |
| 34.5816434 | 0.909090909 | 0.485714286 |
| 35.6666434 | 0.909090909 | 0.457142857 |
| 36.36 | 0.909090909 | 0.428571429 |
| 36.7997042 | 0.909090909 | 0.4 |
| 39.5847042 | 0.909090909 | 0.371428571 |
| 43.2352545 | 0.954545455 | 0.371428571 |
| 45.338376 | 0.954545455 | 0.342857143 |
| 47.4881215 | 0.954545455 | 0.314285714 |
| 50.5473022 | 0.954545455 | 0.285714286 |
| 56.40165 | 1 | 0.285714286 |
| 63.1773603 | 1 | 0.257142857 |
| 67.4824405 | 1 | 0.228571429 |
| 72.5863185 | 1 | 0.2 |
| 76.8594563 | 1 | 0.171428571 |
| 78.6616408 | 1 | 0.142857143 |
| 79.9915684 | 1 | 0.114285714 |
| 84.8574934 | 1 | 0.085714286 |
| 89.555 | 1 | 0.057142857 |
| 91.61775 | 1 | 0.028571429 |
